# Methionine Metabolism in Aging Regulation

**DOI:** 10.1093/geroni/igab046.1759

**Published:** 2021-12-17

**Authors:** Holly Brown-Borg

**Affiliations:** University of North Dakota School of Medicine & Health Sciences, Grand Forks, North Dakota, United States

## Abstract

Aging is the major risk factor for many diseases but the mechanisms are poorly understood. The risk of developing hepatic steatosis increases with age and the health impact of this disease is negative and high. When challenged with high fat diets, long living Ames mice withstand the detrimental metabolic effects that occur in normal mice. We examined transcriptomic and epigenomic profiles of Ames and wild type hepatocytes in the presence or absence of fat to demonstrate that the epigenomic profile drives transcription factor and downstream gene expression resulting in susceptibility or resistance to fatty liver disease. We found that markers of steatosis are related to gene expression in wild type and Ames mice, and dwarf mice retain fewer lipid droplets compared to wild type mice. These studies will provide data to guide our understanding of mechanisms leading to hepatic disease and define factors that provide protection from age-related metabolic disorders.

